# The Clinical Society of Maryland

**Published:** 1893-01

**Authors:** Wm. T. Watson

**Affiliations:** Secretary; 1519 N. Broadway, Baltimore


### THE CLINICAL SOCIETY OF MARYLAND.

Baltimore, November 18th, 1892. The 271st regular meeting was called to order by the president, Dr. William E. Moseley.

Doctors T. C. Gilchrist, Theodore Cooke, Jr. and James McShane, were elected to membership.

DR. S. A. KEENE read a paper on "Personal Experience in the Cholera Epidemic of 1886." Dr. Keene said in part: About the latter part of September, 1866, cholera broke out among some oyster men while dredging in the lower waters of the Chesapeake Bay. The origin was assigned to a vessel hailing from Philadelphia, where the disease had been raging for two or three months. I saw my first patient one midnight. He had been vomiting and purging not more than an hour or two. I believed it to be an ordinary attack of cholera morbus as there was not the slightest suspicion of cholera any where nearer than Philadelphia. I prescribed opium and bismuth, to be followed by a purgative and probably mustard poulticies to the abdomen. Next morning while on my way to see this patient, a messenger met me with a call in another direction. I followed the messenger and when I reached the patient I found him affected similarly to the one I had seen the night before, and from him I learned for the first time that there was a disease prevailing among the oyster men dredging at James' Island, from which they were "dying like sheep," and that he and Slocum, my first patient, becoming alarmed, had left their boats the evening before to come home, about thirty-five miles distance. On the way, Slocum complained of feeling badly, but when they parted, about 10 o'clock, he had not vomited. My second patient had slept well during the night, and had been only aroused early that morning by a desire to evacuate the bowels. I prescribed the same remedies as for my first patient. I hurried to my first patient and found him dying. He died two hours later, within twelve hours from the time he and his companion separated the night before, and within nine hours from my first visit. I returned immediately to my second patient and found that vomiting had come on during my absence and the purging had also increased. He seemed quite prostrated and very anxious. Really I did not know what to do. With a very limited experience, for I had only graduated eighteen months before, all alone in a country place, having just left a corpse and now standing before a most probably prospective one, you may well imagine my feelings. If I had never before appreciated the responsibilities of my profession, it is needless to say that now I realized them all. Opening my little armamentarium and thinking all the while what I should give, I could conjure nothing nor select anything more than what I had already given him. During my perplexity, the patient's old mother suggested that injections of red oak bark tea might be of service. I eagerly accepted the suggestion. The old lady knew how to make and give the injection, so I left to consult my books, promising to return soon. When I reached home there was another call which I found to be a similar case. Within four or five days I received seventeen calls to cholera patients, all of them oyster dredgers and coming from the affected boats. I gathered up my journals and read them as well as I could on my route, while my boy drove. The different authors did not agree any better then than now, and I found no encouragement from my dilemma. Opium seemed to be the only thing agreed upon, but I had tried it and seen it fail. I had already learned that time was valuable and action must be prompt. I was constrained to believe that the tendency to exhaustion could be best met by stimulation, and for that purpose I combined chloroform, tinct. camphor, tinct. capsici, tinct. opii and brandy, of which I gave liberally and frequently. I was pleased with the effect. It not only stimulated the patient, but it relieved the cramps, and, I believe, had a controlling influence over the vomiting. At any rate, sixteen of the seventeen cases recovered. To revert to my second patient: when I returned, I found him much relieved of the purging and in every way better, and by the next morning he was well. I have no doubt but the red oak bark injections were of great benefit to this patient. I adopted it in all of the other cases This little outbreak of cholera, a partial account of which is here given, did not last more than two or three weeks, and, in accord with its peculiar characteristics, the greatest virulence and mortality were in the beginning. The cases I saw were not in the places infected, but were removed to their homes, many miles away. There was but one case in which I had any suspicion of contagion, and that was the wife of one of my patients who had a very slight gastro-intestinal irritation.

DR. W. T. HOWARD, JR., read a paper on "Heart Hypertrophy; an Analysis of 105 Cases from the Autopsy Records of the Johns Hopkins Hospital."

DR. A. C. POLE read a paper entitled, "A Contribution to the Literature of Foreign Bodies in Surgery." On September 7, 1885, G. W., who was assaulted by a crowd of roughs, presented himself to Dr. Pole with an incised wound between the anterior and upper part of the auricle and the temporal bone. The wound was examined and cleaned, and as the foreign body was neither seen nor suspected, the wound was stitched and dressed, and in a few days it was healed by primary union. Two years later the patient had a discharge from his ear and behind a fungus-like growth could be felt what seemed to be a projection of uncovered bone. He was seen by an eminent specialist who attempted at several sittings to remove the "bone" but without avail. Examination from time to time showed that the projecting body was lowering in the auditory canal and was becoming slightly movable. September 22, 1892, the patient was anæsthetized and Dr. Pole removed through the external auditory meatus a piece of dagger blade measuring 31-32 of an inch in length, the point having entered the posterior wall of the canal and penetrated into the mastoid cells to the depth of about 5-8 of an inch. The piece of blade had been in this position for seven years. The patient is now quite well. He has been relieved entirely of severe neuralgic pains from which he had suffered for several years past.

DR. J. M. T. FINNEY related a case of "Severed Fingers Re-applied Seven Hours After Accident with Perfect Union, and Recovery of Motion and Sensation." On January 2, 1890, the patient, a machinist by trade, came to the Johns Hopkins Hospital about half past twelve o'clock, giving the following history. He was a machinist by trade and was running the engine in the absence of the regular engineer, in a tin shop. He went to work about five o'clock that morning, and a little later, while going about a machine used for chopping blocks of tin, he dropped something and while stooping down to pick it up his hand slipped under the knife and the ends of the middle and ring fingers were cut off. The middle finger was cut off just beyond the last joint. The joint was opened. The ring finger was cut off just above the root of the nail This occurred, the man said, about half-past five o'clock. He wrapped up the stumps and went home where his wife covered the wounds with beeswax. He arrived at the hospital at the time previously stated. I asked him where the stumps of the fingers were, and he produced them wrapped up in a piece of newspaper. They were very cold, almost frozen. I placed them in a basin of warm water, using no antiseptic because bichloride or carbolic acid might cause a layer of coagulation necrosis and prevent union. I scrubbed up the stumps of the fingers with a 1-2,000 warm bichloride solution, then I carefully rinsed them off in warm water. This process consumed at least half an hour. Then I took a shaving off the ends of the fingers so as to have a perfectly fresh surface. The stumps were treated in the same manner. The bone was scraped. I sewed them on, using four stitches in each case. I then applied strips of crepe lisse with collodion the whole length of the fingers on each side. These held the several portions in exact apposition. Then I used other strips around the fingers, binding them together, and then applied a palmrr splint and used a large absorbent dressing. He came back in a week and when the dressing was removed the fingers looked very well. I reapplied the dressing and told him to report in another week. Dr. Brockway saw the case on his return at the end of the second week. He took out the stitches and removed the dressing and said that there was no doubt but that the fingers had united and that the man seemed to have sensation at the ends of the fingers, although he thought that this sensation might have been transmitted. The man then disappeared entirely from view. He returned about a month ago with an injury to his other hand. It is difficult to say at first sight which hand was injured. There is a slight motion in the joint which was opened and the sensation in the fingers is perfect.

DR. RANDOLPH WINSLOW: This case of Dr. Finney's calls to my mind a case which I had about fifteen years ago. I was called one day to see a woman who followed the occupa- of an upholstress. She had chopped the end of her thumb off with a hatchet, perhaps a half an hour before I saw her. Upon making inquiry about the missing piece, I was told that it was about the floor somewhere. I hunted it up, cleaned it, put it on with adhesive strips and it is there to this day. It is rather an important matter that we should replace these lost parts and in many cases we will have success. I have a number of times replaced parts which were essentially cut off, attached by a minute portion of skin, with successful union. WM. T. WATSON, Secretary.

1519 N. Broadway, Baltimore.

